# Heart Rate Turbulence Predicts Survival Independently From Severity of Liver Dysfunction in Patients With Cirrhosis

**DOI:** 10.3389/fphys.2020.602456

**Published:** 2020-12-09

**Authors:** Tope Oyelade, Gabriele Canciani, Matteo Bottaro, Marta Zaccaria, Chiara Formentin, Kevin Moore, Sara Montagnese, Ali R. Mani

**Affiliations:** ^1^Institute for Liver and Digestive Health, Division of Medicine, UCL, London, United Kingdom; ^2^School of Medicine, Sapienza University of Rome, Rome, Italy; ^3^Department of Medicine, University of Padova, Padua, Italy

**Keywords:** heart rate turbulence, turbulence onset, cirrhosis, survival, meld, autonomic nervous system, turbulence slope

## Abstract

**Background:**

Reduced heart rate variability (HRV) is an independent predictor of mortality in patients with cirrhosis. However, conventional HRV indices can only be interpreted in individuals with normal sinus rhythm. In patients with recurrent premature ventricular complexes (PVCs), the predictive capacity of conventional HRV indices is compromised. Heart Rate Turbulence (HRT) represents the biphasic change of the heart rate after PVCs. This study was aimed to define whether HRT parameters could predict mortality in cirrhotic patients.

**Materials and Methods:**

24 h electrocardiogram recordings were collected from 40 cirrhotic patients. Turbulence Onset was calculated as HRT indices. The enrolled patients were followed up for 12 months after the recruitment in relation to survival and/or transplantation.

**Results:**

During the follow-up period, 21 patients (52.5%) survived, 12 patients (30%) died and 7 patients (17.5%) had liver transplantation. Turbulence Onset was found to be strongly linked with mortality on Cox regression (Hazard ratio = 1.351, *p* < 0.05). Moreover, Turbulence Onset predicted mortality independently of MELD and Child-Pugh’s Score.

**Conclusion:**

This study provides further evidence of autonomic dysfunction in cirrhosis and suggests that HRT is reliable alternative to HRV in patients with PVCs.

## Introduction

Patients with cirrhosis exhibit systemic manifestations such as autonomic dysfunction which affects adaptation to physiologic and pathologic challenges. The mechanism of autonomic dysfunction in cirrhosis is unknown but several longitudinal studies have shown that autonomic dysfunction is linked with poor prognosis in this patient population (Chan et al., 2017; Bhogal et al., 2019). Autonomic dysfunction can be assessed non-invasively using a variety of methods based on computational analysis of heart rate fluctuations (Chan et al., 2017; Jansen et al., 2018; Bhogal et al., 2019).

Heart rate variability (HRV) reflects the dynamic modulation of heart rate by autonomic nervous system. Reduced HRV is widely reported in cirrhotic patients (Ates et al., 2006; Newton et al., 2006; Mani et al., 2009; Bhogal et al., 2019; Jansen et al., 2019; Satti et al., 2019), particularly, by Bhogal et al. (2019) who demonstrated that the HRV indices can predict mortality in cirrhotic patients, independent of their MELD (Model of End-Stage Liver Dysfunction) score - one of the most used grading system used to predict short term mortality. However, these indices were only linked to mortality in cirrhotic patients with normal sinus rhythm. In cirrhotic patients with abnormal sinus rhythm with recurrent premature ventricular complexes (PVCs) observed on a 24-h ECG recording, the predictive capacity of HRV indices may be challenging.

Heart Rate Turbulence (HRT) is the variation of the length of the cardiac inter-beat intervals after PVCs. In normal conditions, HRT is characterized by a defined pattern: an initial acceleration followed by a deceleration of heart rate (Bauer et al., 2008). HRT was first described by Schmidt et al. in a study that linked the absence of this phenomenon with a higher risk of mortality in post-myocardial infarction patients, independently of other risk factors (Schmidt et al., 1999). From a physiological point of view, the vagus nerve activity seems to play an important role in HRT. Blood pressure drop after the PVC leads to a vagal withdrawal and subsequent heart rate acceleration. In reverse, vagal recruitment seems to provoke the heart rate deceleration leading to the pre-PVC values (Ghuran et al., 2002). Sympathetic excitation during the first phase of post PVC period and sympathetic inhibition during the late phase has also been observed (Segerson et al., 2007). These evidences support the theory that the blood pressure drop after the PVC triggers the baroreflex, which leads both to the vagal modulation of the sinus cycle and to the increase of sympathetic activity in the peripheral tissues. For all these reasons, the physiological background of HRT looks to be strongly determined by autonomic system regulation.

We have recently investigated the prognostic value of physio-markers (e.g., conventional HRV indices and body temperature variability) in a cohort of patients with cirrhosis (Bottaro et al., 2020). However, it rapidly became apparent to us that, most patients exhibit PVCs in their 24-h ECG recording. In these patients, HRT could be considered for assessment of autonomic function. Jansen et al., demonstrated the relationship between two parameters of HRT (Turbulence Onset and Turbulence Slope) and cirrhosis (Jansen et al., 2018). Whereby, the severity of cirrhosis correlated positively and negatively with Turbulence Onset (TO) and Turbulence Slope (TS) respectively (Jansen et al., 2018). Nevertheless, a correlation between HRT indices and mortality in cirrhotic patients was not investigated in that study or any other.

The present study reports our investigations to define whether HRT parameters could predict mortality in cirrhotic patients. We also aimed to understand if HRT parameters have a correlation with mortality independent of Child-Pugh’s and MELD scores.

## Materials and Methods

### Ethics

Ethics approval for the main study (Bottaro et al., 2020) was obtained from the University of Padova Ethics Committee (4169/AO/17). Written informed consent was provided by all patient involved and data was collected and stored appropriately. Patients also gave their consent regarding future use of the collected data.

### Study Population

Sample size calculation: Sample size was calculated to demonstrate an area under the curve of 75% in the ROC curve for predicting mortality using a physiological marker. With the assumption of 45% mortality during 12 months follow-up, 40 participants were required to reach a significance level of 0.05 with a power of 0.80.

Participants: 40 patients with cirrhosis admitted to the Clinica Medica 5 (Padova University Hospital) were approached, consented and enrolled between 6th of April 2017 and 2nd of February 2019. Recruited patients were classified by the etiology of their liver diseases based on clinical, laboratory, radiological and histological results. The severity of liver failure was staged using the Child-Pugh and the Model for End-Stage Liver Disease (MELD) scores. Patients were excluded if they were under 16 years of age; had cirrhosis on a transplanted liver, had atrial fibrillation or implanted pacemaker, severe co-morbidity with short prognosis such as sepsis, a history of neurological or psychiatric disease other than hepatic encephalopathy, active alcohol misuse, or were on psychoactive medication. The mean age of the eligible patients was 64.62 ± 10.4 years.

### Data Collection

24-h electrocardiograph (ECG) recordings suitable for HRT analysis were obtained for research purpose using a wireless Holter recorder (Actiwave Cardio, CamNtech, Cambridge, United Kingdom). Patients were then followed up for 12 months and information was collected on the occurrence of death/liver transplantation. Patients who were transplanted due to liver failure were classed as non-survivors as they were in immediate need of a new liver and wouldn’t survive without transplantation (Bhogal et al., 2019). Patients that could not be followed up for the 12-month period were censored on the date contact was lost. Patients who underwent liver transplantation due to hepatocellular carcinoma were censored on the date of transplantation as the main reason for transplantation was treatment of malignancy and not complications of liver failure.

### Heart Rate Turbulence

Patients having at least one PVC during the 24-h ECG recording were eligible for HRT analysis (Ghuran et al., 2002). Indices of HRT were calculated for each patient using the HRV analysis software (version 1.2, June 2019 release) (Pichot et al., 2016). PVCs were detected using an algorithm developed by Pichot et al. (2016). In brief, PVCs were detected by calculating the prematurity of each beat and its compensatory pause with the mean of the five previous beats as the reference. If the prematurity was >20% and the compensatory pause >120%, then, the beat was considered as a PVC. The indices of HRT measured were Turbulence Onset (TO) and Turbulence Slope (TS). Standard HRT calculation involved the presence of PVCs usually advanced by early 2–3 reduced R-R intervals followed by 10–20 increased R-R intervals (Schmidt et al., 1999; Bauer et al., 2008). TO is the difference between the mean of the two R-R intervals after a PVC and the two R-R interval preceding the PVC divided by the mean of the two RR intervals preceding the PVC [(R-R1 + R-R2) – (R-R-2 + R-R-1)]/(R-R-2 + R-R-1) where R-R-2, R-R-1 are the two R-R intervals before/preceding the PVCs and R-R2, R-R1 are the two R-R intervals immediately after/proceeding the PVCs. Total TO is measured in percentage (%) and is calculated as the mean of the TOs of all the sampled PVCs within a 24-h ECG recording. Physiologically, TO should be negative as R-R-2 + R-R-1 is expected to be higher than R-R2 + R-R1.

TS is the maximum positive regression slopes of any consecutive five points of up to the 20th R-R intervals proceeding/following a PVC. The total TS is measured in ms/R-R and is the overall average of all the TS computed over a 24-h Holter ECG recording. By convention, physiologically normal TS should be positive with a higher TS associated with both significant early reduced and late increase in R-R interval after a PVC (Bauer et al., 2008).

In order to investigate the correlation between HRT and HRV in this cohort of patients, HRV indices were measured by calculating SDNN (standard deviation of inter-beat intervals) in the PVC-free sections of the 24-h ECG using a computational filter (Pichot et al., 2016). Since SDNN is heavily affected by basal heart rate, corrected SDNN (cSDNN) for basal heart rate was calculated using the following formula as descried by Monfredi et al. (2014):

$$\text{cSDNN}=\frac{\text{SDNN}}{{\text{e}}^{-}\frac{\mathrm{Heart}\mathrm{rate}}{58.8}}$$

Other conventional measures of HRV including RMSSD (root mean square of the successive differences of R-R intervals), SDANN (standard deviation of the average R-R intervals calculated over 5 minutes), pNN50 (the proportion of number of pairs of successive R-R intervals that differ by more than 50 ms divided by total number of R-R intervals), Ultra-Low Frequency (ULF), Very-Low Frequency (VLF), Low-Frequency (LF) and High-Frequency (HF) powers were also calculated using HRV analysis software (version 1.2, June 2019 release) (Pichot et al., 2016).

### Statistical and Survival Analysis

To assess the relationship between the indexes of HRT (i.e., TO and TS) and the survival outcome of patients, we performed independent t-test for normally distributed data and Man-Whitney U-test for data not normally distributed. A significant level was determined with a p-value less than 0.05. We performed Cox regression to analyze the effect of HRT parameters on patient’s survival. We calculated Cox regression coefficient (β) and the Hazard Ratio (e^β^). The null hypothesis (β = 0, e^β^ = 1) was tested against a *p*-value of <0.05 calculated by Wald test. ROC curve was used to decide the best HRT indices cut-off points with combined optimum sensitivity and specificity for the prediction of survival. For survival analysis we used the Kaplan-Meier graph, log-rank (Mantel-Cox) test to determine whether the cut-off generated can distinguish the two groups. To estimate the magnitude of differences in HRT indices between the two groups (survivors vs. non-survivors) we used Hedges’g estimation of effect size to compare the TO and TS of the survivors and the non-survivors. SPSS Statistics 20 (IBM Corp., Armonk, New York) was used for statistical analysis.

## Results

### Study Populations

The recruitment and follow-up of patients was initially for an investigation to assess if physio-markers such as body temperature variability and HRV can predict survival in cirrhotic patients (Monfredi et al., 2014). We extended the analysis in this study to investigate the prognostic value of HRT in this cohort. Overall, 40 patients were followed up for up to 12 months post-recruitment. Of these, 30 (75%) were males. As expected, the mean (± SD) of MELD score between survivors (17.82 ± 1.76) and non-survivors (23.76 ± 1.98) was significantly different (*p* = 0.031). The mean (± SD) Child-Pugh scores of the survivor (8.90 ± 0.43) and the non-survivor (10.54 ± 0.58) were also significantly different (p = 0.022). The mean age of all patients was 64 (range 45 to 84) years with no significant difference in mean (± SD) age of the survivor (64.0 ± 2.3) and non-survivor (65.0 ± 2.5) groups (*p* = 0.768). There was also no significant difference in gender proportions between the groups (*P* = 0.637). The demographics and general clinical features of the study population is presented in Table 1.

**TABLE 1:** Demographic and clinical variables in the study population.

Age (years)	64.62 ± 10.4
Gender (male/female)	30/10
Etiology of cirrhosis (number patients)	Alcoholic (17), Viral (10), Metabolic (5), Viral + Alcoholic (6), Viral + metabolic (1), Cryptogenic (1)
Main reason for hospital admission (number of patients)	Hepatic encephalopathy (15), Tense ascites (11), Hepatorenal syndrome (6), Bleeding esophageal varices (1), combination of reasons (7)
MELD	20.4 ± 8.6
Child-Pugh Score	9.6 ± 2.3
Child class (number of patients)	A (3), B (15), C (22)
On admission sodium level (mEq/L) [normal range]	136 ± 6 [135–145]
On admission creatinine level (μmol/L) [normal range]	133 ± 133 [female: 44–97, male: 53–106]
On admission albumin level (g/L) [normal range]	29.0 ± 8.4 [35–55]
On admission bilirubin level (μmol/L) [normal range]	105 ± 128 [5.1–17]
On admission INR [normal range]	1.74 ± 0.65 [0.8–1.1]
On admission erythrocyte sedimentation rate (mm/hr) [normal range]	34 ± 28 [Female: ≤ 20 mm/hr, Male: ≤ 15 mm/hr]
On admission SpO2 (%) [normal range]	98 ± 2 [95–100]
On admission body temperature (°C)	36.7 ± 0.8
On admission hear rate (bpm)	75 ± 14
On admission systolic arterial pressure (mmHg)	128 ± 21
On admission diastolic arterial pressure (mmHg)	70 ± 11
History of hypertension (±)	5/35
History of diabetes (±)	6/34
Beta-blocker (±)	15/25

*The data are expressed as mean ± SD. MELD, model for End-stage Liver disease; INR, International normalized ratio.*

**FIGURE 1:**
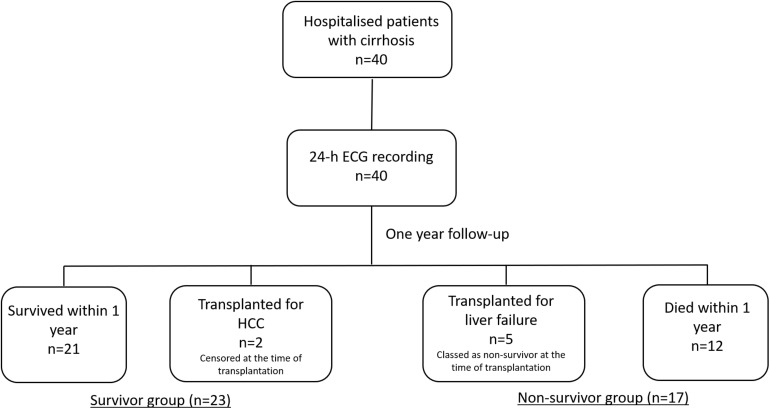
A flowchart of the study protocol. 40 patients with cirrhosis were followed up for one year. Patients who were transplanted due to liver failure were classed as non-survivors as they were in immediate need of a new liver and wouldn’t survive without transplantation. Patients who underwent liver transplantation due to hepatocellular carcinoma were censored on the date of transplantation as the main reason for transplantation was treatment of malignancy and not complications of liver failure.

During the follow up period, 12 (30%) deaths was recorded and 21 (52.5%) survived. The causes of death during 12-month follow-up were sepsis (*n* = 4), hepatorenal syndrome (*n* = 2), hepatocellular carcinoma (*n* = 2), myocardial infarction (*n* = 2), secondary bacterial peritonitis (*n* = 1) and acute alcoholic hepatitis (*n* = 1). Five (12.5%) of the patients had liver transplantation for liver failure while 2 (5%) had liver transplantation due to hepatocellular carcinoma (Figure 1). Patients who underwent transplantation for hepatocellular carcinoma were censored on the date of transplantation. Patients who were transplanted due to liver failure were classed as non-survivors as they were in immediate need of a new liver and wouldn’t survive without transplantation. Therefore, hazard ratio was calculated based on 17 mortality events.

### HRT Indices Between Survivor and Non-survivor

The mean (± SD) number of PVCs during 24-h recording were 27.87 ± 35.47 and 31.88 ± 40.76 in survivors and non-survivors respectively (*p* = 0.7542). There was no significant difference in the means of TS between the survivor and non-survivor. However, TO between the groups showed a significance difference (-0.01 ± 2.6 vs. 1.42 ± 1.3, *p* = 0.03). This is presented in Table 2. The effect size between the two group was observed to be small for TS (Hedges’ g = 0.2) while medium to large in TO (Hedges’ g = 0.7).

**TABLE 2:** The mean Heart Rate Turbulence indices of the study population.

	Survivors	Non-survivors	*p*-value
Study size	23	17	–
TO (%)	−0.01 ± 2.6	1.42 ± 1.3	**0.03**
TS (ms/R-R)	3.83 ± 4.5	2.86 ± 5.4	0.54
No of PVCs in 24 h: Median (range)	7 (1–110)	13 (1–155)	0.742

*TS and TO data are expressed as mean ± SD. Level of significance set at: *p* < 0.05. TO: Turbulence Onset, TS: Turbulence Slope, PVC: Premature Ventricular Contraction. Bolded values mean that they are statistically significant.*

### HRT and Survival

To determine the relationship between HRT and mortality, we performed Cox regression analysis. According to Table 3, of the three indices (TO and TS and PVC number) analyzed, only TO was significantly linked with mortality (Hazard Ratio = 1.351, *p* < 0.05, Table 3). With hazard ratio of 1.351, translating into a 35% increase in mortality for every unit increase in TO.

**TABLE 3:** The predictive effect of age, hepatic dysfunction and indices of Heart Rate Turbulence on 1-year mortality.

	β	SEM	Hazard Ratio	*p*-value
Age	0.007	0.024	1.007	0.778
MELD	0.069	0.027	1.072	**0.009**
Child-Pugh	0.303	0.122	1.345	**0.013**
TO	0.301	0.122	1.351	**0.014**
TS	–0.030	0.069	0.971	0.670
No of PVCs in 24 h	0.000	0.006	1.000	0.987

*Univariate Cox regression analysis is used for calculation of hazard ratio. β is the coefficient of Cox regression analysis. SEM is the standard error of the mean of β, Hazard ratio = Exp (β) = e^β^. TO: Turbulence Onset, TS: Turbulence Slope, PVC: Premature Ventricular Contraction. Bolded values mean that they are statistically significant.*

### HRT Is Independent of Indices of Liver Failure in Predicting Survival

Moving forward, we tested whether the predictive power of TO was independent of MELD and Child-Pugh scores. Cox regression analysis showed that TO significantly predicted mortality independently of MELD and Child-Pugh score (Hazard Ratio TO adjusted for Child-Pugh Score = 1.342, *p* < 0.05 and Hazard Ratio TO adjusted for MELD Score = 1.290, *p* < 0.05). As expected, MELD and Child-Pugh scores also showed a positive association with mortality, with higher scores correlating with poorer prognosis independently of the HRT score (Table 4).

**TABLE 4:** The independence of Turbulence Onset from the MELD score **(A)** and Child-Pugh score **(B)** in predicting mortality in bivariate Cox regression analysis, TO: Turbulence Onset.

(A)	β	SEM	Hazard Ratio	*p*-value
TO	0.254	0.120	1.290	**0.034**
MELD	0.067	0.029	1.069	**0.020**

**(B)**	**β**	**SEM**	**Hazard Ratio**	***p*-value**

TO	0.295	0.138	1.342	**0.033**
Child-Pugh	0.297	0.128	1.346	**0.020**

*Bolded values mean that they are statistically significant.*

### Effect of Beta-Blocker on HRT

Fifteen patients had received a beta blocker for management of portal hypertension. We wondered if taking beta blocker would affect HRT indices (Lin et al., 2004) or survival rates in patients with cirrhosis. Basal heart rate was lower in cirrhotic patients who had received a beta receptor blocking agent (81.8 ± 2.8 vs. 64.4 ± 2.1 beats/min, *p* < 0.000). TO was higher in patients with beta blocker medication (0.03 ± 0.41 vs. 1.54 ± 0.57, *p* < 0.036). However, there was no difference in TS between beta blocker-positive and beta blocker-negative groups (4.19 ± 1.15 vs. 2.11 ± 0.64 ms/R-R, *p* = 0.194). Besides, receiving beta blocker was not associated with an increase in mortality rate after 12-month follow-up (*p* = 0.426). Bivariate Cox regression analysis also showed that TO predicts mortality independently from beta blocker treatment (Hazard ratio of TO adjusted for beta-blocker = 1.347, *p* = 0.02). Bivariate Cox regression analysis also showed that taking beta-blocker is not a predictor of mortality in our cohort (*p* = 0.942).

### Kaplan-Meier Graph for Turbulence Onset

**FIGURE 2:**
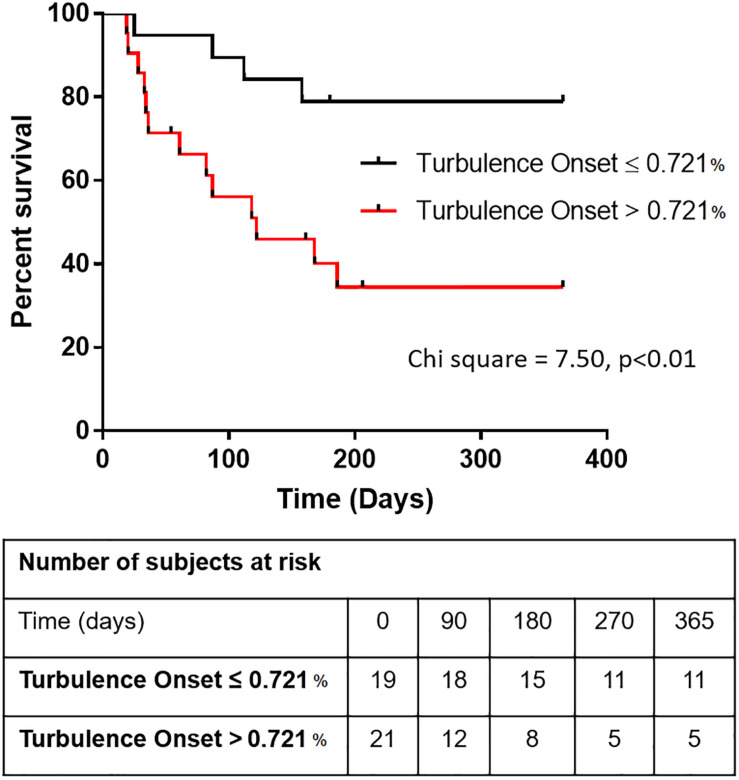
Kaplan-Meier graphs illustrating how Turbulence Onset (TO) can predict survival in patients with cirrhosis. The survival graph depicts the overall survival of cirrhotic patients above and below the cut-off value for Turbulence Onset of 0.721% [Log-rank (Mantel-Cox) test, Chi square = 7.500, *p* < 0.01].

Having shown that TO is significantly predictive of survival in cirrhotic patients independently of MELD, Child-Pugh and beta blocker treatment, Kaplan-Meier graphs were then obtained to further study this relationship. To determine TO cut-off value which presents a balance between sensitivity and specificity in predicting survival, ROC curve analysis was performed. According to the ROC curve, the area under the curve for TO was 72.0 ± 8.1% (*p* = 0.019) with a cut off value of 0.721%. This value distinguished cirrhotic patients with higher risk from patients with lower risk of mortality with a sensitivity of 76.5% and a specificity of 65.2% (Supplementary Appendix 1). This cut-off value significantly discriminated between patients with poor prognosis (TO > 0.721%) and those with higher survival rate (TO ≤ 0.721%) using a Kaplan-Meier survival analysis (Chi-squared = 7.5, *p* = 0.0062, Log-rank Mantel-Cox test; Figure 2).

### Correlation Between HRT and HRV Indices

As shown in Table 5, both TS and TO were markedly correlated with SDNN and cSDNN (measures of total HRV). TS was also correlated with measures of short-term HRV such as pNN50, RMSSD, and HF. In contrast, TO showed significant correlations with measures of long-term HRV including SDANN, ULF and VLF (table 5). There was a significant reduction in SDNN, cSDNN, SDANN, and ULF between survivors and non-survivors (Supplementary Appendix 2-A). Among HRV parameters only SDNN, cSDNN, SDANN, and ULF were predictors of mortality as assessed by univariate Cox regression analysis (Supplementary Appendix 2-B). As shown in Supplementary Appendix 2-B, none of short-term HRV indices could significantly predict mortality.

**TABLE 5:** Correlation between heart rate turbulence indices and heart rate variability indices in the study population.

	TO	TS
SDNN	−0.382*	0.322*
cSDNN	−0.413*	0.410*
RMSSD	–0.085	0.522**
SDANN	−0.386*	0.197
pNN50	–0.087	0.568**
ULF	−0.376*	0.110
VLF	−0.343*	0.180
LF	–0.156	0.255
HF	–0.076	0.462**

TO: Turbulence Onset, TS: Turbulence slope, SDNN: Standard Deviation of inter-beat intervals, cSDNN: SDNN corrected for heart rate ($cSDNN=\frac{SDNN}{e^{-}\frac{Heartrate}{58.8}}$). RMSSD: Root mean square of the successive differences of R-R intervals (a measure of short-term HRV). SDANN: Standard deviation of the average R-R intervals calculated over 5 min (a measure of long-term HRV). pNN50: The proportion of number of pairs of successive RR intervals that differ by more than 50 ms divided by total number of RR intervals. Ultra-Low Frequency (ULF), Very-Low Frequency (VLF), Low-Frequency (LF) and High-Frequency (HF) powers were calculated based on spectral analysis of HRV as described (Pichot et al., 2016). Data are expressed as Pearson’s correlation coefficient. * *P* < 0.05, ** *P* < 0.01 to test the null hypothesis that there is no correlation between the indices (r = 0).

## Discussion

In this study, we report that an index of heart rate turbulence is a strong predictor of survival in patients with liver cirrhosis. Turbulence onset (TO) was discovered among other indices to be significantly correlated with mortality over a 12-month follow-up period. This predictive power was independent of prognostic markers of liver disease severity such as MELD and Child-Pugh scores. Our analysis also showed that although TO was higher in patients who took beta blockers, the prognostic value of TO did not depend on beta blocker treatment.

**FIGURE 3:**
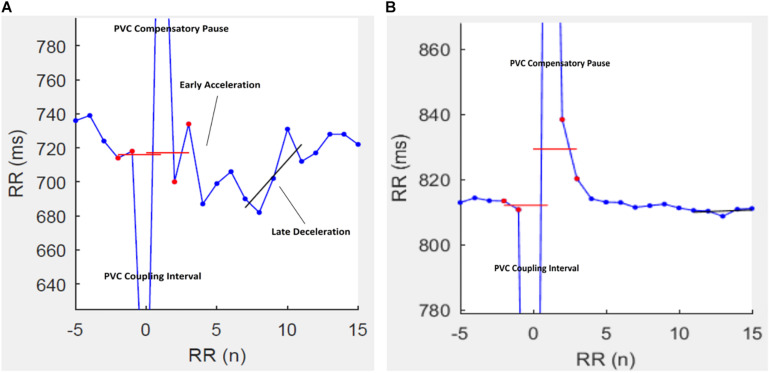
Representative tachograms of heart rate turbulence (HRT) in two patients with cirrhosis. In patients **(A)** HRT is characterized by a post-PVC heart rate acceleration followed rapidly by a deceleration and then a return to the pre-PVC rate. A post-PVC heart rate fade-off in patient **(B)** is characteristic of autonomic dysfunction.

The use of physiological biomarkers (physio-markers) for clinical assessment and prognosis in patients with chronic liver disease has been a topic of interest in the recent years (Fleisher et al., 2000; Coelho et al., 2001; Ates et al., 2006; Newton et al., 2006; Mani et al., 2009; Abrahamovych et al., 2017; Chan et al., 2017; Jansen et al., 2018, 2019; Bhogal et al., 2019; Satti et al., 2019). Much interest has been shown lately especially in the use of indices of HRV as a prognostic tool in various diseases (de Lima et al., 2015; Poliwczak et al., 2019; Urbanik et al., 2019; Braunisch et al., 2020; Yalim et al., 2020; Yamada et al., 2020). This is due to the ease of use and non-invasive nature of these method. However, the presence of PVCs in ECG time-series compromises the viability of HRV indices as a physio-marker. HRT following PVCs is a common physiological feature characterized by proceeding increased and then reduced heart rates followed by return to pre-ectopic rate (Figure 3) (Newton et al., 2006). Previously, HRT have been proposed to be controlled by the coordination of the both arms of the autonomic nervous system, thus, indices of HRT have been purported as a good marker to assess autonomic neuropathy in disease settings (Schmidt et al., 1999; Jansen et al., 2018). This is interesting as some indices of HRV, such as the short-term and long-term HRV have also been linked to the autonomic dysfunction in cirrhosis (Frith and Newton, 2009; Mani et al., 2009; Jansen et al., 2019). However, because of the dependence of conventional HRV methods on normal sinus rhythm, HRT turbulence can serve as a good alternative option especially since PVCs are expected in recorded time series (Kennedy et al., 1985). Interestingly, we observed that TO was significantly correlated with the measures of long-term HRV while TS was associated with short-term HRV indices. Short-term HRV measures are mechanistically linked with the modulation of heart rate by the respiratory cycles; a physiological phenomenon is mainly mediated by the vagus nerve. Thus, it is not surprising to observe that short-term HRV indices are correlated with TS (a measure of vagus-mediated recovery of heart rate following a PVC). Only TO was an independent predictor of mortality in our cohort. This finding corroborates with previous reports showing that only long-term measures of HRV are independent predictors of mortality in patients with cirrhosis (Bhogal et al., 2019; Satti et al., 2019).

Although the present study indicates that impaired HRT following PVCs is a predictor of mortality in patients with cirrhosis, the reason for this observation is not well understood. It can be speculated that impaired HRT or reduced HRV in cirrhosis may reflect the presence of cirrhotic cardiomyopathy. Indeed, cirrhotic patients often present with subclinical cardiomyopathy (Ruíz-del-Árbol et al., 2013; Møller and Lee, 2018). However, there have been no report to show a significant correlation between indices of autonomic cardiac control (i.e., HRT, HRV) and clinical measures of cirrhotic cardiomyopathy in cirrhotic patients. Therefore, whether changes in HRT reported in cirrhosis reflects the presence of cirrhotic cardiomyopathy remains unclear and awaits further investigations. According to studies on animal models of cirrhosis, pharmacological interventions (e.g., nitric oxide synthase inhibitors and low molecular thiols) that improve cardiac function in cirrhotic rats do not improve cardiac autonomic dysfunction in these animal models (Mani et al., 2006). This suggests that cirrhotic cardiomyopathy and autonomic dysfunction in cirrhosis probably have different mechanism and should not be considered as the same phenomenon.

Cardiac autonomic dysfunction is a hallmark of cirrhosis (Chan et al., 2017; Jansen et al., 2018; Bhogal et al., 2019) and might be helpful for patients’ prognostication for liver transplant allocation procedure. However, before suggesting the use of HRT as a prognostic factor for organ allocation, it is crucial to know whether autonomic dysfunction responds positively to liver transplant. It appears that autonomic dysfunction is improved only partially following transplantation in patients with cirrhosis (Chan et al., 2017; Bhogal et al., 2018). Thus, measures of autonomic dysfunction (e.g., impaired HRT) might be considered as a co-morbidity factor in the process of organ allocation (Bhogal et al., 2018). In our study, among 17 non-survivors, 2 patients died of myocardial infarction which accounts for 11.7% of non-survivors. When we looked at their HRT indices, both patients had a TO higher than the cut off value (0.734 and 1.016). This suggests that HRT may also identify subgroup of patients who suffer from co-morbidities (e.g., ischemic heart disease) not directly associated with liver failure. In order to test whether our results have been confounded by this co-morbidity, we retrospectively excluded those two cases who died of myocardial infarction and only included patients who died as a result of complications directly associated with cirrhosis (Supplementary Appendix 3). The results showed that TO remains a significant predictor of survival independently of MELD and Child-Pugh’s scores even after excluding patients whose cause of death was myocardial infarction (Supplementary Appendices 3, 4 and Supplementary Appendix 5). Although this goes along with our report that HRT predicts survival independently of liver dysfunction in cirrhosis, further studies are required to elucidate potential clinical applications of the link between impaired HRT and survival in cirrhosis.

In the present study, we determined a cut-off value for TO for prediction of mortality in cirrhosis and did not report our results based on previously reported cut-off values (e.g., 0% for TO and 2.5 ms/R-R for TS) and categories of HRT (0-1-2) which were originally shown to be predictive for mortality in patients with acute myocardial infarction (MI) (Schmidt et al., 1999; Ghuran et al., 2002; Bauer et al., 2008). Although both acute MI and cirrhosis exhibit autonomic dysfunction, distinctive components of autonomic function appears to be involved in these two illnesses (Tapanainen et al., 2002; Bottaro et al., 2020). Following acute MI, both TO and TS can predict mortality (Schmidt et al., 1999; Ghuran et al., 2002). However, in patients with cirrhosis TS is not significantly different between survivors and non-survivors and only TO is a predictor of mortality. Such a difference between acute MI and cirrhosis has also been shown in studies where non-linear measures of HRV were used for determination of prognosis (Tapanainen et al., 2002; Bottaro et al., 2020). It appears that short-term fractal scaling (α1) of HRV is a powerful predictor of mortality among patients surviving an acute myocardial infarction while only long-term fractal scaling (α2) predicts mortality in patients with cirrhosis (Tapanainen et al., 2002; Bottaro et al., 2020). Based on these reports, it is not surprising to observe that prognostic models that have been developed for acute MI may not be applicable to cirrhosis. As shown in Supplementary Appendix 6, we applied categories HRT (0-1-2) for patients with cirrhosis and observed that although there is a significant difference in mortality between HRT = 0 and HRT 1 or 2, cirrhotic patients with HRT = 1 exhibit higher rate of mortality in comparison with HRT = 2 in the first 6 months of their follow up (Appendix 6). TS is not a predictor of death in patients with cirrhosis and this may explain why HRT categories that are based on combination of TO and TS are not suitable for survival analysis in cirrhosis. In our study, patients in HRT category 1 (either TO > 0% or TS < 2.5 ms/R-R) predominantly had TO ≥ 0%. In fact, 8 out of 12 patients in HRT category 1 had TO > 0% while 4 out of 12 had TS > 2.5 ms/R-R.

### Limitations

Because data were collected from patients admitted to the hospital for decompensated liver disease, the result cannot be extrapolated to outpatients with less severe (compensated) cases of cirrhosis. Also, the sample size, although sufficiently powerful, represents a single Center, random sub-population and may not represent the full spectrum of patient with liver cirrhosis. For further validation of use of HRT as a prognostic physio-marker we recommend the use of a larger and more diverse patient population validated with healthy control group probably involving multiple and varied clinical settings.

In our samples, 13 patients had less than six PVCs in their 24-h ECGs. Some investigators have suggested calculating HRT when more than five PVCs are available as the reliability of PVC tachograms may decrease with the lower numbers of PVCs (Bauer et al., 2008). In the present study, patients having at least one PVC during the 24-h ECG recording were considered for HRT analysis to include as many patients as possible in the survival analysis. Future studies can include this criterion (number of PVC > 5) for calculation of HRT indices in a larger dataset.

The complexity of clinical environment in hospitalized patients did not allow us to study circadian variations in HRT parameters in our study. It is well established that cirrhosis is associated with circadian abnormalities (Montagnese et al., 2010; Garrido et al., 2017). Previous reports have also shown that there are circadian oscillations in HRT indices particularly in the TS (Chen, 2011). Future studies in controlled laboratory settings may indicate a difference in circadian variations of HRV/HRT indices in survivor and non-survivor groups.

Although we initially aimed to assess cardiac autonomic function in the presence of cardiac arrhythmia in patients with cirrhosis, our results can only be applied when PVCs disrupt normal sinus rhythms. The main challenge for assessment of autonomic function from R-R intervals is the presence of atrial fibrillation. Atrial fibrillation limits the interpretability of both HRV and HRT methods and requires further attention in future investigations.

## Conclusion

In conclusion, we report in this pilot study that TO, an index of heart rate turbulence, as a physio-marker may predict survival in cirrhotic patients. We also report a cut-off of TO that significantly distinguish patients at higher risk of mortality within 12 months.

## Data Availability Statement

The original contributions presented in the study are included in the manuscript/Supplementary Material, further inquiries can be directed to the corresponding author/s.

## Ethics Statement

The studies involving human participants were reviewed and approved by the University of Padova Ethics Committee (4169/AO/17). The patients/participants provided their written informed consent to participate in this study.

## Author Contributions

TO, GC, SM, and AM performed conceptualization. MB, CF, SM, and AM performed data curation. GC, TO, MZ, and AM performed formal analysis. SM, KM, and AM performed supervision. TO and GC wrote original draft of the manuscript. TO, CF, KM, SM, and AM wrote, review and editing the manuscript. All authors contributed to the article and approved the submitted version.

## Conflict of Interest

The authors declare that the research was conducted in the absence of any commercial or financial relationships that could be construed as a potential conflict of interest.

## Supplementary Material

Supplementary Appendix 1 ROC curve for prediction of mortality using Turbulence Onset (TO). Area under the curve for TO = 0.720 (*p* = 0.019).

Supplementary Appendix 2 Heart rate variability (HRV) indices calculated from 24-h ECG recordings of patients with cirrhosis. **(A)** Comparison of survivors and non-survivors. **(B)** The predictive effect of HRV indices on 1-year mortality in patients with cirrhosis.

Supplementary Appendix 3 The mean Heart Rate Turbulence indices of the study population after excluding two patients who died due to myocardial infarction. The data are expressed as mean ± SD. TO: Turbulence Onset, TS: Turbulence Slope. PVC: Premature Ventricular Complex.

Supplementary Appendix 4 The independence of Turbulence Onset from the MELD score in predicting mortality excluding two patients who died due to myocardial infarction. Bivariate Cox regression analysis were used for calculation of hazard ratio. TO: Turbulence Onset. β is the coefficient of Cox regression analysis. SEM is the standard error of the mean of β, Hazard ratio = Exp (β) = e^β^. TO: Turbulence Onset.

Supplementary Appendix 5 The independence of Turbulence Onset from the Child-Pugh score in predicting mortality excluding two patients who died due to myocardial infarction. Bivariate Cox regression analysis were used for calculation of hazard ratio. TO: Turbulence Onset. β is the coefficient of Cox regression analysis. SEM is the standard error of the mean of β, Hazard ratio = Exp (β) = e^β^. TO: Turbulence Onset.

Supplementary Appendix 6 Kaplan-Meier graphs illustrating three categories of heart rate turbulence (HRT) based on criteria established for prediction of mortality in patients with acute myocardial infarction by Barthel et al. (Circulation 2003; 108:1221–1226). Within the context of acute myocardial infarction, the cut-off value for Turbulence Onset (TO) and Turbulence Slope (TS) is 0% and 2.5 ms/R-R respectively. Patients were classified into the following HRT categories: category 0 if both TO and TS were normal (TO < 0% and TS > 2.5 ms/R-R), category 1 if either TO or TS was abnormal, or category 2 if both TO and TS were abnormal (Chi square = 6.26, *p* = 0.044).

Supplementary Appendix 7 The STROBE checklist.
