# Supplementary material for: Heart Rate Turbulence Predicts Survival Independently From Severity of Liver Dysfunction in Patients With Cirrhosis
Source: Front Physiol. 2020 Dec 9;11:602456. doi: 10.3389/fphys.2020.602456 (PMC7755978; doi:10.3389/fphys.2020.602456)

**Supporting information**

**S1 fig** ROC curve for prediction of mortality using Turbulence Onset (TO). Area under the curve for TO = 0.720 (p=0.019).


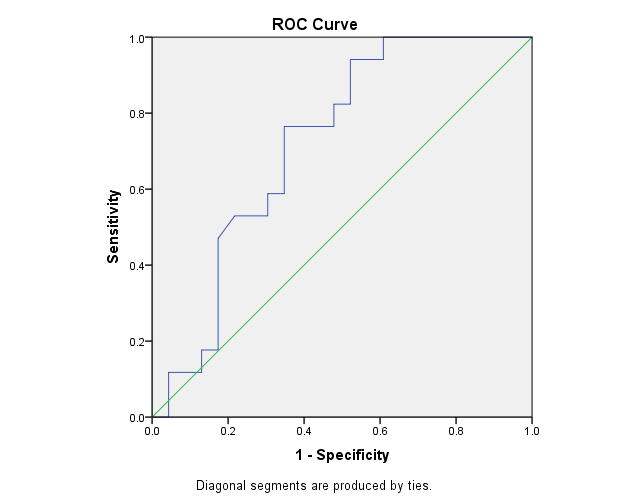

Supplement: Supplementary Appendix 1 — ROC curve for prediction of mortality using Turbulence Onset (TO). Area under the curve for TO = 0.720 (p = 0.019). [file Table_1.DOCX]
