# Supplementary material for: Heart Rate Turbulence Predicts Survival Independently From Severity of Liver Dysfunction in Patients With Cirrhosis
Source: Front Physiol. 2020 Dec 9;11:602456. doi: 10.3389/fphys.2020.602456 (PMC7755978; doi:10.3389/fphys.2020.602456)
Supplement: Supplementary Appendix 2 — Heart rate variability (HRV) indices calculated from 24-h ECG recordings of patients with cirrhosis. (A) Comparison of survivors and non-survivors. (B) The predictive effect of HRV indices on 1-year mortality in patients with cirrhosis. [file Table_2.docx]

**Supporting information**

**Appendix 2:** Heart rate variability (HRV) indices calculated from 24-hr ECG recordings of patients with cirrhosis. A. Comparison of survivors and non-survivors. B. The predictive effect of HRV indices on 1-year mortality in patients with cirrhosis.

**A.** Comparison of HRV indices between survivors and non-survivors. Data are shown as mean ± SEM.

|  | **Survivors** | **Non-survivors** | **p-value** |
| --- | --- | --- | --- |
| **SDNN (ms)** | 86.2±6.2 | 63.4±6.1 | **0.014** |
| **cSDNN** | 317±23 | 236±24 | **0.020** |
| **RMSSD (ms)** | 25.1±3.7 | 34.3±6.7 | 0.235 |
| **SDANN (ms)** | 74.7±6.0 | 49.2±4.2 | **0.002** |
| **pNN50 (%)** | 7.4±2.3 | 13.3±4.4 | 0.207 |
| **ULF (ms^2^)** | 4908±1487 | 1621±290 | **0.040** |
| **VLF (ms^2^)** | 954±206 | 610±195 | 0.242 |
| **LF (ms^2^)** | 326±102 | 386±187 | 0.768 |
| **HF (ms^2^)** | 194±60 | 326±131 | 0.370 |

**B.** The predictive effect of HRV indices calculated from 24-h ECG on 1-year mortality in hospitalised patients with cirrhosis. Univariate Cox regression analysis was used for calculation of hazard ratio.

|  | **β** | **SEM** | **Hazard Ratio** | **p-value** |
| --- | --- | --- | --- | --- |
| **SDNN** | -0.026 | 0.010 | 0.975 | **0.010** |
| **cSDNN** | -0.007 | 0.003 | 0.993 | **0.015** |
| **RMSSD** | 0.015 | 0.011 | 1.016 | 0.151 |
| **SDANN** | -0.037 | 0.012 | 0.964 | **0.003** |
| **pNN50** | 0.024 | 0.016 | 1.024 | 0.129 |
| **ULF** | -0.000 | 0.000 | 1.000 | **0.044** |
| **VLF** | 0.000 | 0.000 | 1.000 | 0.241 |
| **LF** | 0.000 | 0.000 | 1.000 | 0.587 |
| **HF** | 0.001 | 0.001 | 1.001 | 0.134 |

β is the coefficient of Cox regression analysis. SEM is the standard error of the mean of β, Hazard ratio =𝐸𝑥𝑝 (β) = 𝑒^β^. SDNN: Standard Deviation of inter-beat intervals, cSDNN: SDNN corrected for heart rate ($\mathbf{cSDNN=}\frac{\mathbf{SDNN}}{\mathbf{e}^{\mathbf{-}}\frac{\mathbf{Heart rate}}{\mathbf{58.8}}}$). RMSSD: Root mean square of the successive differences of RR intervals (a measure of short-term HRV). SDANN: Standard deviation of the average NN intervals calculated over short periods, usually 5 minutes (a measure of long-term HRV). pNN50: The proportion of number of pairs of successive RR intervals that differ by more than 50 ms divided by total number of RR intervals. Ultra-Low Frequency (ULF), Very-Low Frequency (VLF), Low-Frequency (LF) and High-Frequency (HF) bands were calculated based on spectral analysis of HRV.
