# Supplementary material for: Heart Rate Turbulence Predicts Survival Independently From Severity of Liver Dysfunction in Patients With Cirrhosis
Source: Front Physiol. 2020 Dec 9;11:602456. doi: 10.3389/fphys.2020.602456 (PMC7755978; doi:10.3389/fphys.2020.602456)
Supplement: Supplementary Appendix 3 — The mean Heart Rate Turbulence indices of the study population after excluding two patients who died due to myocardial infarction. The data are expressed as mean ± SD. TO: Turbulence Onset, TS: Turbulence Slope. PVC: Premature Ventricular Complex. [file Table_3.DOCX]

**Supporting information**

**Appendix 3:** The mean Heart Rate Turbulence indices of the study population after excluding two patients who died due to myocardial infarction. The data are expressed as mean ± SD. TO: Turbulence Onset, TS: Turbulence Slope. PVC: Premature Ventricular Complex.

|  | **Survivors** | **Non-Survivors** | **p-value** |
| --- | --- | --- | --- |
| **Study Size** | 23 | 15 | - |
| **TO** | -0.01±2.6 | 1.49±1.3 | **0.025** |
| **TS** | 3.83±4.5 | 3.15±5.6 | 0.685 |
| **No of PVCs in 24 hours** | 27.87±35.47 | 25.20±27.04 | 0.806 |
