# Supplementary material for: Heart Rate Turbulence Predicts Survival Independently From Severity of Liver Dysfunction in Patients With Cirrhosis
Source: Front Physiol. 2020 Dec 9;11:602456. doi: 10.3389/fphys.2020.602456 (PMC7755978; doi:10.3389/fphys.2020.602456)
Supplement: Supplementary Appendix 4 — The independence of Turbulence Onset from the MELD score in predicting mortality excluding two patients who died due to myocardial infarction. Bivariate Cox regression analysis were used for calculation of hazard ratio. TO: Turbulence Onset. β is the coefficient of Cox regression analysis. SEM is the standard error of the mean of β, Hazard ratio = Exp (β) = eβ. TO: Turbulence Onset. [file Table_4.DOCX]

**Supporting information**

**Appendix 4:** The independence of Turbulence Onset from the MELD score in predicting mortality excluding two patients who died due to myocardial infarction. Bivariate Cox regression analysis were used for calculation of hazard ratio. TO: Turbulence Onset*.* β is the coefficient of Cox regression analysis. SEM is the standard error of the mean of β, Hazard ratio =𝐸𝑥𝑝 (β) = 𝑒^β^. TO: Turbulence Onset.

|  | **β** | **SEM** | **Hazard Ratio** | **p-value** |
| --- | --- | --- | --- | --- |
| **TO** | 0.267 | 0.127 | 1.306 | **0.036** |
| **MELD** | 0.063 | 0.031 | 1.065 | **0.042** |
