# Supplementary material for: Heart Rate Turbulence Predicts Survival Independently From Severity of Liver Dysfunction in Patients With Cirrhosis
Source: Front Physiol. 2020 Dec 9;11:602456. doi: 10.3389/fphys.2020.602456 (PMC7755978; doi:10.3389/fphys.2020.602456)
Supplement: Supplementary Appendix 5 — The independence of Turbulence Onset from the Child-Pugh score in predicting mortality excluding two patients who died due to myocardial infarction. Bivariate Cox regression analysis were used for calculation of hazard ratio. TO: Turbulence Onset. β is the coefficient of Cox regression analysis. SEM is the standard error of the mean of β, Hazard ratio = Exp (β) = eβ. TO: Turbulence Onset. [file Table_5.DOCX]

**Supporting information**

**Appendix 5:** The independence of Turbulence Onset from the Child-Pugh score in predicting mortality excluding two patients who died due to myocardial infarction. Bivariate Cox regression analysis were used for calculation of hazard ratio. TO: Turbulence Onset*.* β is the coefficient of Cox regression analysis. SEM is the standard error of the mean of β, Hazard ratio =𝐸𝑥𝑝 (β) = 𝑒^β^. TO: Turbulence Onset.

|  | **β** | **SEM** | **Hazard Ratio** | **p-value** |
| --- | --- | --- | --- | --- |
| **TO** | 0.301 | 0.142 | 1.351 | **0.034** |
| **Child-Pugh** | 0.260 | 0.132 | 1.296 | **0.048** |
