# Supplementary material for: Heart Rate Turbulence Predicts Survival Independently From Severity of Liver Dysfunction in Patients With Cirrhosis
Source: Front Physiol. 2020 Dec 9;11:602456. doi: 10.3389/fphys.2020.602456 (PMC7755978; doi:10.3389/fphys.2020.602456)
Supplement: Supplementary Appendix 6 — Kaplan-Meier graphs illustrating three categories of heart rate turbulence (HRT) based on criteria established for prediction of mortality in patients with acute myocardial infarction by Barthel et al. (Circulation 2003; 108:1221–1226). Within the context of acute myocardial infarction, the cut-off value for Turbulence Onset (TO) and Turbulence Slope (TS) is 0% and 2.5 ms/R-R respectively. Patients were classified into the following HRT categories: category 0 if both TO and TS were normal (TO < 0% and TS > 2.5 ms/R-R), category 1 if either TO or TS was abnormal, or category 2 if both TO and TS were abnormal (Chi square = 6.26, p = 0.044). [file Table_6.DOCX]

**Supporting information**

**Appendix 6:** Kaplan-Meier graphs illustrating three categories of heart rate turbulence (HRT) based on criteria established for prediction of mortality in patients with acute myocardial infarction by Barthel et al. (Circulation 2003; 108:1221-1226). Within the context of acute myocardial infarction, the cut-off value for Turbulence Onset (TO) and Turbulence Slope (TS) is 0% and 2.5 ms/RR respectively. Patients were classified into the following HRT categories: category 0 if both TO and TS were normal (TO<0% and TS >2.5 ms/RR), category 1 if either TO or TS was abnormal, or category 2 if both TO and TS were abnormal (Chi square = 6.26, p=0.044).

**
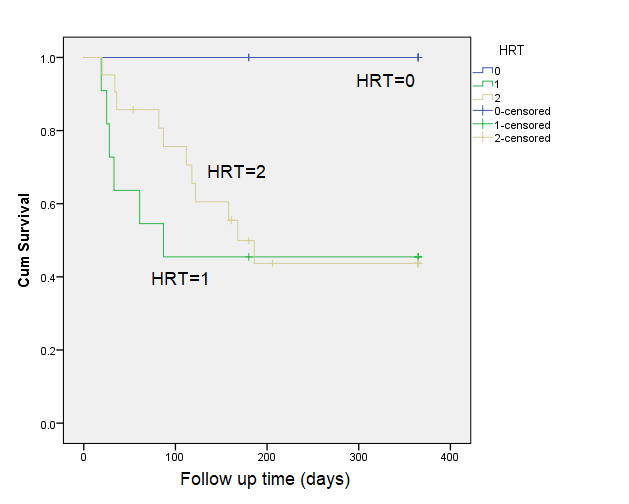
**
