# Supplementary material for: Heart Rate Turbulence Predicts Survival Independently From Severity of Liver Dysfunction in Patients With Cirrhosis
Source: Front Physiol. 2020 Dec 9;11:602456. doi: 10.3389/fphys.2020.602456 (PMC7755978; doi:10.3389/fphys.2020.602456)
Supplement: Supplementary Appendix 7 — The STROBE checklist. [file Table_7.DOC]

STROBE Checklist — Heart rate turbulence predicts survival independently from severity of liver dysfunction in patients with cirrhosis

|  | Item No | Recommendation | Manuscript Page | Reported in Manuscript Section |
| --- | --- | --- | --- | --- |
| **Title and abstract** | 1 | (*a*) Indicate the study’s design with a commonly used term in the title or the abstract | 1 | Title /Abstract |
|  |  | (*b*) Provide in the abstract an informative and balanced summary of what was done and what was found | 2 | Abstract |
| Introduction | | | | |
| Background/rationale | 2 | Explain the scientific background and rationale for the investigation being reported | 2-3 | Introduction |
| Objectives | 3 | State specific objectives, including any prespecified hypotheses | 3 | Introduction |
| Methods | | | | |
| Study design | 4 | Present key elements of study design early in the paper | 3 | Ethics, Study Population |
| Setting | 5 | Describe the setting, locations, and relevant dates, including periods of recruitment, exposure, follow-up, and data collection | 3-4 | Study Population, Data Collection |
| Participants | 6 | (*a*) *Cohort study*—Give the eligibility criteria, and the sources and methods of selection of participants. Describe methods of follow-up | 3-4 | Study Population, Data Collection |
| Variables | 7 | Clearly define all outcomes, exposures, predictors, potential confounders, and effect modifiers. Give diagnostic criteria, if applicable | 3-4 | Study Population, Data Collection |
| Data sources/ measurement | 8* | For each variable of interest, give sources of data and details of methods of assessment (measurement). Describe comparability of assessment methods if there is more than one group | 3-4 | Data Collection, Heart Rate Turbulence, Statistical and Survival Analysis |
| Bias | 9 | Describe any efforts to address potential sources of bias | 3 | Study Population |
| Study size | 10 | Explain how the study size was arrived at | 3 | Study Population |
| Quantitative variables | 11 | Explain how quantitative variables were handled in the analyses. If applicable, describe which groupings were chosen and why | 4 | Statistical and Survival Analysis |
| Statistical methods | 12 | (*a*) Describe all statistical methods, including those used to control for confounding | 4 | Heart Rate Turbulence, Statistical and Survival Analysis |
|  |  | (*b*) Describe any methods used to examine subgroups and interactions | 4 | Heart Rate Turbulence, Statistical and Survival Analysis |
|  |  | (*c*) Explain how missing data were addressed | NA | No Missing Data |
|  |  | (*d*) *Cohort study*—If applicable, explain how loss to follow-up was addressed | 3-4 | Data Collection |
|  |  | (*e*) Describe any sensitivity analyses | 4 | Heart Rate Turbulence |
| **Results** | | | | |
| Participants | 13* | (a) Report numbers of individuals at each stage of study—eg numbers potentially eligible, examined for eligibility, confirmed eligible, included in the study, completing follow-up, and analysed | 5 | Study Population |
|  |  | (b) Give reasons for non-participation at each stage | N/A | Study Population |
|  |  | (c) Consider use of a flow diagram | . | Figure 1 |
| Descriptive data | 14* | (a) Give characteristics of study participants (eg demographic, clinical, social) and information on exposures and potential confounders | 5 | Study Population |
|  |  | (b) Indicate number of participants with missing data for each variable of interest | NA | No Missing Data |
|  |  | (c) *Cohort study*—Summarise follow-up time (eg, average and total amount) | 5 | Study Population |
| Outcome data | 15* | *Cohort study*—Report numbers of outcome events or summary measures over time | 5 | Study Population |
| Main results | 16 | (*a*) Give unadjusted estimates and, if applicable, confounder-adjusted estimates and their precision (eg, 95% confidence interval). Make clear which confounders were adjusted for and why they were included | 5-6 | HRT Indices between Survivor and Non-survivor, HRT and Survival, HRT is independent of indices of liver failure in predicting survival, Effect of Beta-Blocker on HRT, Kaplan-Meier graph for Turbulence Onset |
|  |  | (*b*) Report category boundaries when continuous variables were categorized | 6 | Kaplan-Meier graph for Turbulence Onset |
|  |  | (*c*) If relevant, consider translating estimates of relative risk into absolute risk for a meaningful time period |  | Figure 2 |
| Other analyses | 17 | Report other analyses done—eg analyses of subgroups and interactions, and sensitivity analyses | 6 | Correlation between HRT and HRV indices |
| **Discussion** | | | | |
| Key results | 18 | Summarise key results with reference to study objectives | 6 | Discussion |
| Limitations | 19 | Discuss limitations of the study, taking into account sources of potential bias or imprecision. Discuss both direction and magnitude of any potential bias | 7 | Limitations |
| Interpretation | 20 | Give a cautious overall interpretation of results considering objectives, limitations, multiplicity of analyses, results from similar studies, and other relevant evidence | 6-7 | Discussion |
| Generalisability | 21 | Discuss the generalisability (external validity) of the study results | 7-9 | Discussion |
| Other information | | | | |
| Funding | 22 | Give the source of funding and the role of the funders for the present study and, if applicable, for the original study on which the present article is based | 12 | Funding |
